# Anthropologists' views on race, ancestry, and genetics

**DOI:** 10.1002/ajpa.23120

**Published:** 2016-11-22

**Authors:** Jennifer K. Wagner, Joon‐Ho Yu, Jayne O. Ifekwunigwe, Tanya M. Harrell, Michael J. Bamshad, Charmaine D. Royal

**Affiliations:** ^1^ Geisinger Health SystemDanvillePennsylvania17822; ^2^ University of WashingtonSeattleWashington98195; ^3^ Duke UniversityDurhamNorth Carolina27708; ^4^ Fred Hutchinson Cancer Research CenterSeattleWashington98109

**Keywords:** diversity, racism, survey

## Abstract

Controversies over race conceptualizations have been ongoing for centuries and have been shaped, in part, by anthropologists.

**Objective:**

To assess anthropologists' views on race, genetics, and ancestry.

**Methods:**

In 2012 a broad national survey of anthropologists examined prevailing views on race, ancestry, and genetics.

**Results:**

Results demonstrate consensus that there are no human biological races and recognition that race exists as lived social experiences that can have important effects on health.

**Discussion:**

Racial privilege affects anthropologists' views on race, underscoring the importance that anthropologists be vigilant of biases in the profession and practice. Anthropologists must mitigate racial biases in society wherever they might be lurking and quash any sociopolitical attempts to normalize or promote racist rhetoric, sentiment, and behavior.

## 1 Introduction

Heated controversies over the use, (mis)appropriation, harmful effects, and rejection of race continue to make regular headlines. The continued use of race concepts in genetic research was described recently as “problematic at best and harmful at worst” (Yudell, Roberts, DeSalle, & Tishkoff, 2016, p. 564). Two years ago Dobbs (2014) denounced Nicholas Wade's “A Troublesome Inheritance” (2014)—a book in which Wade, using a combination of circular logic, speculation, and “just‐so” stories, made unsubstantiated claims about the genetic basis for three major human races—and described it as “a deeply flawed, deceptive and dangerous book.” One hundred forty‐three leading human geneticists admonished Wade for his “misappropriation” of their research and rejected the idea that their work substantiated any of Wade's racial claims (Coop et al., 2014). While use of race is problematic, some researchers have indicated when it comes to eliminating racial injustices, “‘there are much bigger fish to fry’ than scrubbing race as a biological category” (Begley, 2016). Moreover, simple substitution of terms (e.g., race, population, and ancestry) is not a panacea, because each scientific question requires selection of an appropriate lens through which it can be answered.

Anthropologists, regardless of prevailing perspective on race, are (or have the capacity to be) highly influential on common conceptualizations of race (Smedley, 1993). Nearly 40 years ago, Lieberman and Reynolds (1978, p. 33) conducted an empirical investigation to determine the prevailing views on race among anthropologists at the time, having noted belief of the existence of human races was “widespread among scientists” generally. Lieberman and Reynolds (1978) examined whether prevailing beliefs on race among physical anthropologists were distinguishable from scientists generally, studied the sociocultural influences on physical anthropologists' perspectives on race, and reported results from a survey of physical anthropologists' levels of agreement with statements about race drawn from the literature. The nine statements used by Lieberman and Reynolds in their study to classify “lumpers” (i.e., those who do not believe races exist), “splitters” (i.e., those who believe races exist), and “intermediate” lumpers or splitters are shown in Table 1. Regardless of whether lumpers or splitters and notwithstanding varying perspectives on the existence of race, Lieberman and Reynolds noted most physical anthropologists were “equalitarian in orientation and liberal in outlook” (Lieberman & Reynolds, 1978, p. 338). Their results showed (1) anthropologists from privileged sociocultural backgrounds (labeled “overdogs”) tended to share the then dominant view on the existence of races and, thus, tended to be splitters and (2) anthropologists from marginalized sociocultural backgrounds (labeled “underdogs”) tended to be lumpers, rejecting the existence of human races.

**Table 1:** Statements examined by Lieberman and Reynolds (1978)

1. “Races are the taxonomic unit below the species level, and if such units are not called race, ‘it still has exactly the same taxonomic meaning.’”
2. “Races vary from populations ‘differing only in that frequencies of a few genes to those grouping have been totally isolated for tens of thousands of years and are at the least incipient species.’”
3. “Clines (gradations) exist but it is necessary to distinguish clines between subspecific populations and clines within subspecific populations. Interracial clines are found in intermediate populations between subspecific populations or races.”
4. “Biological variability exists but ‘this variability does not conform to the discrete packages labeled races.’”
5. “So‐called racial characteristics are not ‘transmitted as complexes.’”
6. “Human differentiation is the result of natural selection forces which operate in ecological zones and such forces and their zones do not coincide with population boundaries. Furthermore, different selective forces may operate in overlapping ecological zones. Thus, ‘geographic distributions of more than one trait have no necessary correlation.’”
7. “Races do not exist because isolation of groups has been infrequent; populations have always interbred.”
8. “Boundaries between what have been called ‘races’ are completely arbitrary, depending primarily upon the wishes of the classifier.”
9. “No races exist now or ever did.”

A subsequent survey in 1985 showed belief in race divided physical anthropologists roughly in half (Lieberman & Reynolds, 1996). While scholars have periodically revisited this topic and noted the “growing awareness of the meaninglessness of racial taxonomy” (Cartmill, 1998, p. 659), as recently as 2003 there was not yet sufficient evidence to conclude a “dramatic recent rejection” of race within the subfield (Cartmill & Brown, 2003, p. 115). By 2009, however, general consensus among leading biological anthropologists on a number of areas was reported, including that “[r]ace is not an accurate or productive way to describe human biological variation” (Edgar & Hunley, 2009, p. 2). Indeed, more nuanced views about race among anthropologists and areas of study have emerged (e.g., Goodman, 2013). In 2012, we decided it appropriate to re‐examine views of anthropologists across all subfields to better understand current prevailing views on race, ancestry, and genetics.^1^ Here, we present that study to inform broader efforts to move scientists “beyond race” and to encourage—as we articulated elsewhere^2^—anthropology's embrace of a holistic, anti‐racist approach.

## 2 Methods

### 2.1 Survey design

A 53‐item survey focused on race, ancestry, and genetics was designed and administered using SurveyMonkey (Palo Alto, CA). Participants were shown a series of statements and asked to rate levels of agreement or disagreement using a five‐point scale. Statements (shown in the left column of Table 2) were organized into five sections covering four themes: statements about science (two sections), statements about medicine, statements about social and societal issues, and common statements about race. Statements previously studied by Lieberman and Reynolds (1978) were used as inspiration for some statements, and four statements were re‐examined verbatim (statements #4, 5, 8, and 9 in Table 1). The survey ended with questions regarding the participant's sex, age, ancestry, race and ethnicity as per the U.S. Census, education level, anthropological subfield, employment sector, country of residence and work, and familiarity (i.e., experience or interest) with genetic ancestry tests. Each section enabled participants to provide comments.

**Table 2:** Statements and summary of responses from professional anthropologists

**Statements about science**	**Common response**	**Correlation**
1. The human population may be subdivided into biological races.	Strongly disagree or disagree (86%)	0.11 **<.00010** 1,909
2. Racial categories are determined by biology.	Strongly disagree or disagree (88%)	0.11 **<.00010** 1,903
3. There are discrete biological boundaries among races.	Strongly disagree or disagree (93%)	0.14 **<.00010** 1,868
4. Biological variability exists but this variability does not conform to the discrete packages labeled races.	Strongly agree or agree (89%)	−0.035 .12 1,894
5. Boundaries between what have been called races are completely arbitrary, depending primarily upon the wishes of the classifier.	Strongly agree or agree (69%)	0.0014 .95 1,898
6. Continental population categories—Africans, Asians, Europeans—are the same as standard anthropological racial classifications.	Strongly disagree or disagree (73%)	0.070 **.0025** 1,872
7. Continental population categories—Africans, Asians, Europeans—are useful for examining genetic relationships (i.e., relatedness) among people.	Strongly disagree or disagree (38%); Strongly agree or agree (33%)	0.21 **<.00010** 1,879
8. Race—as defined by the U.S. Office of Management and Budget (i.e., census categories)—is a useful proxy for ancestry.	Strongly disagree or disagree (73%)	0.043 .057 1,890
9. Genetic ancestry—inferred from genetic markers—rather than race, is a better proxy for genetic relationships among sub‐Saharan Africans, Asians, Europeans, Pacific Islanders, and Native Americans.	Strongly agree or agree (75%)	0.18 **<.00010** 1,887
10. The distributions of physical traits overlap among races.	Strongly agree or agree (89%)	0.016 .48 1,883
11. So‐called racial characteristics are not transmitted as complexes.	Strongly agree or agree (67%)	−0.082 **.00050** 1,814
12. Genetic differences between racial groups explain most biological differences between individuals of different races.	Strongly disagree or disagree (72%)	0.095 **<.00010** 1,888
13. Genetic differences between racial groups explain most behavioral differences between individuals of different races.	Strongly disagree or disagree (95%)	0.091 **<.00010** 1,893
14. Most anthropologists believe that humans may be subdivided into biological races.	Strongly disagree or disagree (85%)	0.094 **<.00010** 1,898
15. Most anthropologists believe that categorizing human groups by race has no biological basis.	Strongly agree or agree (74%)	−0.083 **.00030** 1,896
16. Most anthropologists believe that categorizing human groups by race has no genetic basis.	Strongly agree or agree (61%)	−0.12 **<.00010** 1,877
17. The use of the term ‘race’ to describe human groups should be discontinued.	Strongly agree or agree (71%)	−0.027 .24 1,888
18. The term “race,” as used to describe human groups, should be replaced by a more appropriate and precise term.	Strongly agree or agree (71%)	0.026 .26 1,878
19. Most genetic variants among sub‐Saharan Africans, East Asians, and Western Europeans are shared.	Strongly agree or agree (67%)	−0.036 .13 1,830
20. Most common genetic variants (i.e., alleles with a frequency of >5%) are shared among sub‐Saharan Africans, East Asians, and Western Europeans.	Strongly agree or agree (66%)	0.00086 .97 1,815
21. Genetic variation data may be used to cluster racially ascribed people into groups of continental origin.	Strongly disagree or disagree (37%); Strongly agree or agree (29%)	0.18 **<.00010** 1,843
22. Self‐identified race in the U.S. highly corresponds to genetic inferences of ancestry.	Strongly disagree or disagree (67%)	0.084 **.00030** 1,876
**Statements about medicine**	**Common response**	**Correlation**
1. Race influences health.	Strongly disagree or disagree (42%); Strongly agree or agree (41%)	0.015 .53 1,865
2. Race, in so far as it corresponds with genetic ancestry, influences health.	Strongly agree or agree (47%)	0.14 **<.00010** 1,867
3. Genetic differences between races explain health disparities.	Strongly disagree or disagree (71%)	0.19 **<.00010** 1,847
4. Categorizing individuals by race is important for biomedical research.	Strongly disagree or disagree (58%)	0.12 **<.00010** 1,851
5. Anthropologists should understand key concepts about the relationship among race, genetics and health.	Strongly agree or agree (79%)	0.051 **.03** 1,869
6. Health professionals should understand key concepts about the relationship among race, genetics and health.	Strongly agree or agree (80%)	0.067 **.0037** 1,876
7. The development of medicines for targeted racial groups is a necessary step toward the achievement of personalized medicine.	Strongly disagree or disagree (58%)	0.16 **<.00010** 1,867
8. Race should be considered in diagnosing certain conditions or diseases.	Strongly disagree or disagree (51%)	0.086 **.00020** 1,857
9. Genetic ancestry should be considered in diagnosing certain conditions or diseases.	Strongly agree or agree (79%)	0.14 **<.00010** 1,882
10. Race should be considered in treating certain conditions or diseases.	Strongly disagree or disagree (53%)	0.083 **.00030** 1,850
11. Genetic ancestry should be considered in treating certain conditions or diseases.	Strongly agree or agree (77%)	0.14 **<.00010** 1,885
**Statements about social and societal issues**	**Common response**	**Correlation**
1. Commercial genetic ancestry testing provides a good assessment of a person's ancestry.	Strongly disagree or disagree (39%)	0.28 **<.00010** 1,804
2. Genetic ancestry testing is purely recreational.	Strongly disagree or disagree (33%); Strongly agree or agree (33%)	−0.23 **<.00010** 1,811
3. Genetic ancestry testing does not warrant all the critique it is receiving from the anthropology community.	Strongly disagree or disagree (43%)	0.23 **<.00010** 1,779
4. Genetic ancestry testing is considered by the industry's consumers as purely recreational.	Strongly disagree or disagree (48%)	0.052 **.030** 1,776
5. Consumers value genetic ancestry testing for non‐medical reasons.	Strongly agree or agree (73%)	0.078 **.0010** 1,780
6. Commercial genetic ancestry testing in the U.S. is subject to specific, federal regulation.	Strongly disagree or disagree (39%)	0.0073 .76 1,699
7. Commercial genetic ancestry testing in the U.S. should be subject to specific, federal regulation.	Strongly agree or agree (53%)	−0.054 **.024** 1,760
8. Criticisms of genetic ancestry testing are overstated compared to the actual risks and limitations of the tests.	Strongly disagree or disagree (30%); Strongly agree or agree (16%)	0.20 **<.00010** 1,747
9. Genetic ancestry testing should not be used in U.S. criminal investigations.	Strongly agree or agree (49%)	−0.19 **<.00010** 1,777
10. Genetic ancestry testing should be used by U.S. law enforcement when evaluating conflicting eyewitness descriptions.	Strongly disagree or disagree (61%)	0.15 **<.00010** 1,783
11. Genetic ancestry testing should be considered when agencies place children in foster and adoptive families.	Strongly disagree or disagree (82%)	0.061 .**0094** 1,810
12. Genetic ancestry testing should be used by universities to verify racial or ethnic identity of applicants.	Strongly disagree or disagree (90%)	0.070 **.0030** 1,821
13. Genetic ancestry testing reinforces biological conceptions of race.	Strongly agree or agree (50%)	−0.25 **<.00010** 1,785
14. Genetic ancestry testing undermines biological conceptions of race.	Strongly disagree or disagree (36%); Strongly agree or agree (28%)	0.18 **<.00010** 1,785
**Common statements**	**Common response**	**Correlation**
1. Races don't exist.	Strongly agree or agree (59%)	−0.059 **.011** 1,854
2. No races exist now or ever did.	Strongly agree or agree (53%)	−0.078 **.00080** 1,840
3. Race has no biological basis.	Strongly agree or agree (71%)	−0.16 **<.00010** 1,853
4. Race is biologically meaningless.	Strongly agree or agree (73%)	−.016 **<.00010** 1,867
5. Race has no genetic basis.	Strongly agree or agree (65%)	−0.16 **<.00010** 1,852
6. Race has no biological influence on health.	Strongly agree or agree (49%)	−0.15 **<.00010** 1,832

*Note*. Left Column: Statements about Race used in Survey. Survey asked respondents to indicate their level of agreement with statements using a five‐point Likert Scale (1 = strongly agree; 5 = strongly disagree). Statements were organized into four themes: science, medicine, society, and common statements. Middle Column: Red shading indicates general disagreement, blue shading indicates general agreement, and green shading indicates respondents are divided. Dark shading indicates the level met or exceeded 75% of responses (super‐majority). Right Column: Spearman Correlation Coefficients between familiarity with genetic ancestry inference and individual responses to statements are shown with *p* values at 95% confidence interval and number of observations. Bold font denotes statistically significant correlations.

### 2.2 Sampling strategy and recruitment

Anthropologists of all subfields were the target population for this survey. Because the American Anthropological Association (AAA) is the largest organization of anthropologists and includes members from diverse subfields, the sampling strategy focused on individuals connected to the AAA. Researchers used OutWit Hub (OutWit Technologies, www.outwit.com) to digitally capture (or “scrape”) email addresses from the member and meeting attendee pages of the AAA website between October 5 and October 12, 2012. Survey invitations were emailed directly to 41,231 scraped email addresses with the presumption that account holders were anthropologists. Responses were collected from March 5 until August 30, 2013.

### 2.3 Methodological limitations

There are notable limitations to this methodological approach. While the AAA has wide coverage across subfields, the sampled views of AAA members and meeting attendees might not be representative of the views of all anthropologists or those of any specific subfield. Stratified sampling of anthropologists in the main subfields using targeted recruitment of specialized professional organizations or AAA sections would have been preferred for drawing comparisons among the subfields and reducing sampling error. Generalizability is less of a concern for assessment of views of cultural anthropologists, who constitute an overwhelming majority in the AAA and are adequately represented in the surveyed population.^3^ However, there are many reasons to expect significant self‐selection bias in sampling the AAA membership or meeting attendees to assess views of biological anthropologists on any issue and, specifically, race. For example, the 2010 AAA Executive Board decision to remove references to the word “science” from its long‐range plan statement and the perceived marginalization of anthropologists in subfields rooted in science undoubtedly influenced whether—in 2012 when this survey was administered—anthropologists aligned themselves with or distanced themselves from the AAA (e.g., Glenn, 2010; Lende, 2010a, 2010b; Wood, 2010). While more than 50 years ago Wolf (1964) described anthropology as “the most scientific of the humanities and the most humanistic of the sciences,” the 2010 incident renewed debates over whether anthropology is or values science and drew renewed attention to waxing and waning rifts between subfields. AAA members and AAA meeting attendees who self‐identify as biological anthropologists might hold considerably different perspectives on race from self‐identifying biological anthropologists outside of that sampling frame. This potential source of selection bias error prevents robust comparative analyses between subfields; nevertheless, these data provide an important glimpse of recent perspectives on race held by anthropologists generally.

## 3 Results

A total of 3,286 participants completed the survey. Participant characteristics are summarized in Tables 3 and 4. Eighty‐two percent (82%, *N* = 1,918) of the respondents were professional anthropologists as opposed to students (*N* = 423, 18%). We limit our discussion to perspectives of professionals in order to facilitate a more direct comparison to the previous work by Lieberman and Reynolds (1978). Cultural anthropologists outnumber other subfields in this survey (with cultural anthropologists representing 54% of the professional anthropologists), and academics outnumber those in other sectors (representing 84% of the professional anthropologists). A total of 888 respondents provided free text written comments, some of which we have qualitatively analyzed and reported elsewhere.^4^

**Table 3:** Survey participant characteristics

Professional experience, N (%)	*N*	%
Archaeological Anthropologist	342	12.36
Cultural Anthropologist	1,032	37.28
Linguistic Anthropologist	108	3.90
Medical Anthropologist	235	8.49
Physical/Biological Anthropologist	201	7.26
Anthropology Student or Trainee	440	15.90
Other	410	14.81
**Work Environment, *N* (%)**		
Academic	2,208	81.06
Government	141	5.18
Private Industry	375	13.77
**Place of Residence, *N* (%)**		
Within the U.S.	2,190	80.49
Outside of the U.S.	531	19.51
**Sex, *N* (%)**		
Female	1,700	62.41
Male	1,024	37.59
**Age (Years)**		
Mean	44.7 (SD=15.4)	
Range	Range 19–100 (IQR=32,57)	
**Race (U.S. Census Categories), *N* (%)**		
American Indian or Alaska Native	68	3.00
Asian	70	3.09
Black, African American	97	4.29
Native Hawaiian or Other Pacific Islander	6	0.27
White	1,688	74.59
Other	334	14.76
**Ethnicity (U.S. Census Categories), *N* (%)**		
Hispanic/Latino	204	9.02
Non‐Hispanic/Latino	2,057	90.98

*Note*. Due to item non‐response, number of respondents does not always sum to *N* = 3,286. Most items with responses are *N* = 2,724.

**Table 4:** Survey participant characteristics: professionals only

Professional experience, N (%)	*N*	%
Archaeological Anthropologist	342	17.83
Cultural Anthropologist	1,032	53.81
Linguistic Anthropologist	108	5.63
Medical Anthropologist	235	12.25
Physical/Biological Anthropologist	201	10.48
**Work Environment, *N* (%)**		
Academic	1,596	83.78
Government	90	4.72
Private Industry	219	11.50
**Place of Residence, *N* (%)**		
Within the U.S.	1,499	79.44
Outside of the U.S.	388	20.56
**Sex, *N* (%)**		
Female	1,156	61.00
Male	739	39.00
**Age (years)**		
Mean	47.4 (SD = 14.6)	
Range	21–100 (IQR = 35,60)	
**Race (U.S. Census Categories), *N* (%)**		
American Indian or Alaska Native	40	2.57
Asian	40	2.57
Black, African American	51	3.28
Native Hawaiian or Other Pacific Islander	3	0.19
White	1,182	75.96
Other	240	15.42
**Ethnicity (U.S. Census Categories), *N* (%)**		
Hispanic/Latino	141	9.04
Non‐Hispanic/Latino	1,418	90.96

*Note*. Due to item nonresponse, the number of professional respondents does not always sum to *N* = 1,918.

Table 2 (middle column) provides a summary of the reported levels of agreement and disagreement with statements on race. Only six of the 53 statements reflect undecided perspectives or lack of general consensus among professional anthropologists (including, e.g., whether race influences health and whether genetic ancestry testing undermines biological conceptions of race). Fourteen statements evoked unified responses from a super‐majority, with professional anthropologists (a) rejecting the idea that humans can be subdivided into biological races; the idea that races are biologically determined; the existence of discrete boundaries among races; and the use of genetic ancestry when making child placement or college admission decisions and (b) accepting the existence of biological variation; overlapping trait distributions; the superiority of genetic ancestry over race as a proxy for genetic relationships between peoples; the importance of understanding the relationship among race, genetics and health; and the need to take genetic ancestry into account when diagnosing and treating certain conditions. Informal analysis of the data did not reveal any noticeable differences in perspectives between subfields.

Comparing our results directly with those of Lieberman and Reynolds (1978), we see a sizable shift in agreement with the statement “No races exist now or ever did.” As shown in Table 5, while only a minority of respondents (17%) agreed with that statement in 1978, a majority of respondents (53%) agreed with that statement in 2013. Agreement with this statement is consistent across subfields. Given the academic and public discourses on genetic ancestry testing and concerns about its potential reification of race, a separate analysis was performed to determine whether familiarity with genetic ancestry testing (i.e., a combined testing item that is the sum of the three items—having obtained a genetic ancestry test, interest in getting one, or used genetic ancestry inference in research—with possible scores 0, 1, 2, and 3) was correlated with levels of agreement with the statements about race. Familiarity with genetic ancestry testing was significantly correlated with 43 of the 53 statements (81%), as shown in the right column of Table 2. While a closer examination of perspectives of biological anthropologists not affiliated with AAA or attending AAA meetings is needed, our data (including those shown in Table 6) suggest biological anthropologists and particularly those with familiarity with genetic ancestry testing might be more willing to acknowledge that race—not understood as a biologically‐defined category but, rather, as a socially‐defined category and as a lived experience of structural and institutional racism—can have important effects on health.

**Table 5:** Comparison of current results with those reported by Lieberman and Reynolds (1978)

**Statement**	**Percent of respondents in agreement from Lieberman and Reynold's (1978) data**	**Percent of respondents in agreement from our 2013 data**	**Net change (1978–2013)**
“No races exist now or ever did”	17%	53%	+36%
“Biological variability exists but this variability does not conform to the discrete packages labeled races”	79%	89%	+10%

*Note*. Data drawn from Table 1 (displaying levels of agreement by 141 respondents classified as lumpers, intermediate lumpers, intermediate splitters, and splitters) of Lieberman and Reynolds (1978).

**Table 6:** Comparison of levels of agreement with statements between biological and other types of anthropologists and between those with and without experience with genetic ancestry inference

Statement	Percent of BIO ANTH respondents in agreement	Percent of NON‐BIO ANTH respondents in agreement	Percent of respondents WITH EXPERIENCE in agreement	Percent of respondents WITHOUT EXPERIENCE in agreement
“No races exist now or ever did”	47%	53%	54%	49%
“Race has no biological influence on health”	37%	50%	41%	51%

To reexamine Lieberman and Reynolds (1978) analysis of lumpers and splitters within the context of overdogs and underdogs, we examined four privilege groups (white males, white females, non‐white males, and non‐white females) with regard to their agreement with the four verbatim statements previously noted. Chi‐square tests for the four contingency tables yielded two significant results as shown in Tables 7 and 8 (non‐significant results are shown in Tables 9 and 10). There is a significant difference (*p* = .0109) between privilege groups' agreement with the statement that “variability exists but does not conform to discrete categories,” with white males and females generally appearing less likely to be splitters than non‐white males and females. There is also a significant difference (*p* = .0199) with the privilege groups' agreement with the statement “no races exist now or ever did,” with white males and females—but particularly white males—less likely to be lumpers than non‐white males and females.

**Table 7:** Chi‐square table for “Biological variability exists but this variability does not conform to the discrete packages labeled races”

Frequency row percentage	Lumpers	Splitters	Intermediate	Total
Non‐white females	200	12	20	232
	86.21	5.17	8.62	
Non‐white males	117	13	3	133
	87.97	9.77	2.26	
White females	668	34	29	731
	91.38	4.65	3.97	
White males	392	20	23	435
	90.11	4.60	5.29	
Total	1,377	79	75	1,531

*Note*. *N* = 1,531; item non‐response = 387; Chi‐square statistic DF = 6, value 16.5953, *p* = .0109.

**Table 8:** Chi‐square table for “No races exist now or ever did”

Frequency row percentage	Lumpers	Splitters	Intermediate	Total
Non‐white females	132	59	34	225
	58.67	26.22	15.11	
Non‐white males	72	33	24	129
	55.81	25.58	18.60	
White females	364	214	134	712
	51.12	30.06	18.82	
White males	184	148	85	417
	44.12	35.49	20.38	
Total	752	454	277	1,483

*Note*. *N* = 1483; item non‐response = 435; Chi‐square statistic DF = 6, value 15.0501, *p* = .0199.

**Table 9:** Chi‐square table for “Boundaries between what have been called races are completely arbitrary, depending primarily upon the wishes of the classifier”

Frequency row percentage	Lumpers	Splitters	Intermediate	Total
Non‐White Females	175	30	23	228
	76.75	13.16	10.09	
Non‐White Males	92	24	18	134
	68.66	17.91	13.43	
White Females	507	120	104	713
	69.36	16.42	14.23	
White Males	287	74	76	437
	65.68	16.93	17.39	
Total	1061	248	221	1530

*Note. N*=1530; Item non‐response = 388; Chi‐Square Statistic DF = 6, value 10.1147, *p* = 0.1199.

**Table 10:** Chi‐square table for “So‐called racial characteristics are not transmitted as complexes”

Frequency row percentage	Lumpers	Splitters	Intermediate	Total
Non‐White Females	141	15	64	220
	64.09	6.82	29.09	
Non‐White Males	77	15	35	127
	60.63	11.81	27.56	
White Females	479	48	172	699
	68.53	6.87	24.61	
White Males	291	33	98	422
	68.96	7.82	23.22	
Total	988	111	369	1468

*Note. N*=1468; Item non‐response = 450; Chi‐Square Statistic DF = 6, value 7.5398, *p* = 0.2738.

## 4 Discussion

Our data indicate there has been a “dramatic rejection” of race concepts among professional anthropologists regardless of subfield. We observed consensus that there are no human biological races and recognition that race exists but as lived social experiences that can have important effects on health. As such, anthropologists agree that it is important to understand the relationships among race, genetics, and health.

The data also revealed a correlation between familiarity with genetic ancestry testing (gauged as personal or professional experience with or interest in genetic ancestry testing) and perspectives on race; however, causal connections are not decipherable with data available. In addition, the data confirm the persistence of privilege effects on perspectives of race: while the four varying groups of privilege examined share similar perspectives on the arbitrariness of boundaries and transmission of racial characteristics, perspectives on the existence of human races and variation conforming to discrete categories differ between groups of privilege, with overdogs (here, white males and white females) generally more likely to be splitters rather than lumpers compared to underdogs (here, non‐white males and non‐white females). This distinction has serious power and equity implications given the distribution of privileged splitter perspectives across the subfields and their influence on the direction of research programs, funding allocations, training, and public understandings of race. These findings reinforce previous calls (e.g., Brodkin, Morgen, & Hutchinson, 2011; McGranahan & Rizvi, 2016; Yelvington et al., 2015) for anthropology to be more cognizant of the privileged spaces within its study and practice, to be vigilant in its efforts to eliminate racial biases, to become more inclusive, and to promote solidarity within the discipline as well as outside of it.

While here we used quantitative analysis to revisit the framework of elite splitters and marginalized lumpers, we have elsewhere proposed a more nuanced interpretation of anthropologists' current views on race.^5^ Based on our studies, anthropologists are more aptly describable as “squatters” (i.e., those who maintain race is not biologically meaningful), “shifters” (i.e., those who maintain race is not biologically meaningful but is a social reality), and “straddlers” (i.e., those who recognize the significance and relevance of both biologically informed and sociocultural conceptualizations of race). Because of anthropology's influence on concepts and understanding of race and its position as “the most scientific of the humanities and the most humanistic of the sciences,” anthropologists have an opportunity and moral responsibility to take aggressive steps to mitigate racial biases in society wherever they might be lurking and quash any sociopolitical attempts to normalize or promote racist rhetoric, sentiment, and behavior. Accordingly, anthropologists should critically examine how squatters, shifters, and straddlers affect ways in which research on human variation is conducted and communicated as well as the impact these views have on popular views of race and effectiveness at alleviating experiences of racism. For example, a laudable effort to improve public understanding of race and the “new anthropological synthesis on race” as recognized by Yolanda Moses is the “Race: Are we so different?” project (AAA). Yet even those behind the project recognized early on the limitations of “whiteness” (e.g., core museum staffs continue to be dominated by white males) (Garfinkle & Goodman, 2007).

Racial inequalities persist in and out of the academy and are “deeply woven into the fabric of our social institutions” such that racism is found in individual and collective biases and prejudices as well as in the organizational behaviors that continue “to index race and promote racially unequal outcomes” (Brodkin et al., 2011, p. 547). New mechanisms to combat disparate impacts are gaining support, underscoring the urgency with which anthropologists must take stronger public stances on race and racism in its modern forms. One of these is the recently issued Final Rule (DHHS, 2016) for Section 1557 of the Patient Protection and Affordable Care Act, which has an overarching goal to eliminate racial (and other) disparities in health programs and activities and provides, for the first time, a private right of action for not only intentional discrimination but also actions with discriminatory effects. Because (1) the Final Rule, which took effect July 18, 2016, contains a new and broad definition of “national origin” and (2) the Final Rule is intended to apply to research as well as delivery of healthcare (DHHS, 2016; at 31385), it is incumbent upon anthropologists to be leaders in a better public understanding of race, in the robust ascertainment of the sources of these disparate impacts, and in the deliberative design of appropriate remedial actions to correct them.

In addition, as ancestry and other concepts are increasingly used to frame human differences, anthropologists should revise official position statements on race—e.g., the most recent statements by the AAA (AAA, 1998) and American Association of Physical Anthropologists (AAPA, 1996) from which “ancestry” is wholly absent—to ensure their continued relevance and preempt the public's dismissal of terminological shifts as simply semantics or “political correctness.”

## Footnotes

1 We also assessed the perspectives of professional geneticists, which we plan to report separately as “Attitudes of Genetic Professionals on Race, Ancestry, and Genetics.”

2 Authors, unpublished manuscript/under peer‐review as “Reconstructing Race: A Qualitative Analysis of How Anthropologists Socially Construct Race.”

3 In 1998, it was reported that 70% of AAA membership was comprised of cultural anthropologists.

4 See Footnote 2.

5 See Footnote 2.
